# Generating DNA sequence data with limited resources for molecular biology: Lessons from a barcoding project in Indonesia

**DOI:** 10.1002/aps3.1167

**Published:** 2018-07-13

**Authors:** Gillian H. Dean, Rani Asmarayani, Marlina Ardiyani, Yessi Santika, Teguh Triono, Sarah Mathews, Campbell O. Webb

**Affiliations:** ^1^ Department of Botany University of British Columbia Vancouver V6T1Z4 British Columbia Canada; ^2^ Herbarium Bogoriense Botany Division Research Center for Biology Indonesian Institute of Sciences (LIPI) Cibinong 16911 Bogor West Java Indonesia; ^3^ Present address: Department of Biology University of Missouri–St. Louis St. Louis Missouri 63121 USA; ^4^ Present address: Zoological Society of London (ZSL) Indonesia Program Bogor 16128 Indonesia; ^5^ Arnold Arboretum of Harvard University Boston Massachusetts 02131 USA; ^6^ Present address: CSIRO Australian National Herbarium Canberra Australian Capital Territory 2601 Australia; ^7^ Present address: University of Alaska Museum of the North Fairbanks Alaska 99775 USA

**Keywords:** DNA barcoding, DNA sequence data, limited resources, molecular biology

## Abstract

The advent of the DNA sequencing age has led to a revolution in biology. The rapid and cost‐effective generation of high‐quality sequence data has transformed many fields, including those focused on discovering species and surveying biodiversity, monitoring movement of biological materials, forensic biology, and disease diagnostics. There is a need to build capacity to generate useful sequence data in countries with limited historical access to laboratory resources, so that researchers can benefit from the advantages offered by these data. Commonly used molecular techniques such as DNA extraction, PCR, and DNA sequencing are within the reach of small laboratories in many countries, with the main obstacles to successful implementation being lack of funding and limited practical experience. Here we describe a successful approach that we developed to obtain DNA sequence data during a small DNA barcoding project in Indonesia.

DNA barcoding allows the identification of specimens via DNA amplification and sequencing and provides a useful complement to morphology‐based identification methods in that it is rapid, needs only a small amount of tissue from any stage of the life cycle, and can be performed without extensive knowledge of the organisms (Hebert et al., 2003). The increasing ease and decreasing costs of obtaining DNA sequence data has accelerated advances in systematics, taxonomy, community ecology, and conservation (reviewed in Kress et al., 2015); food and wildlife forensics (reviewed in Staats et al., 2016); monitoring of agricultural pests and invasive species (Ashfaq and Hebert, 2016); and a myriad of human health applications including identification of parasites and disease vectors (Ondrejicka et al., 2014).

In particular, DNA barcoding has become an increasingly important means to aid efforts to catalog biodiversity, and large consortia affiliated under the International Barcode of Life Project (iBOL; http://ibol.org/) are working toward this goal. Although these organizations have engaged with local partners in biodiversity‐rich regions, smaller local barcoding projects still play an important role in contributing to global barcoding initiatives by facilitating the collection of specimens from less accessible locations and filling in gaps for the larger initiatives (Borisenko et al., 2009). Even if in‐country molecular biology is impossible, local scientists can send tissue samples to the Canadian Centre for DNA Barcoding (CCDB; http://www.ccdb.ca), which is part of the iBOL initiative, for DNA extraction and PCR amplification. As described above, the generation of DNA barcodes has the potential to develop many useful resources for the various stakeholders in these countries, such as identifying species listed in the Convention on International Trade of Endangered Species (CITES; Lahaye et al., 2008) and determining authenticity of traditional Chinese medicines (Han et al., 2016).

There is often a disconnect between the locations where the organisms occur and where the sequence data are generated. Most of the world's biodiversity is found in countries that have less well‐developed scientific research infrastructure, whereas DNA sequence data typically have been generated in countries with relatively low levels of terrestrial biodiversity but well‐established infrastructure and a highly trained workforce. There are, however, strong reasons for generating data in the originating countries. The Convention on Biological Diversity (CBD; https://www.cbd.int/) and the Nagoya Protocol on Access and Benefit Sharing lay out a framework for access to genetic resources and benefit sharing (Davis and Borisenko, 2017). Governments of biodiversity‐rich countries have imposed restrictions to limit access to their genetic resources to varying degrees, meaning that in some countries most or all of the molecular biology work must be done in the source country. Beyond the letter of the law, there are also strong ethical and social reasons (in terms of international friendship and collaboration) for foreign scientists to share their expertise and support local efforts. By performing lab work in the country of collection, and fully sharing data, results, and authorship with local scientists, foreign scientists act as true collaborators. The resulting trust is both an investment by the foreign scientists in their own future research opportunities, and a gesture of goodwill that promotes successful science for all involved (Vernooy et al., 2010).

In our experience, major barriers to in‐country work are lack of practical experience using the techniques required to generate high‐quality DNA sequence data, insufficient funding coupled with higher costs for reagents, and a lack of infrastructure. The wider aim of our project was to lay the groundwork for an open access digital flora of Gunung Palung National Park, West Kalimantan, Indonesia, that will include DNA barcode data, complete collection information, taxonomic determinations, and high‐quality photographs. We carried out a pilot study (October 2008 to October 2010) in the Molecular Systematics Laboratory at the Herbarium Bogoriense, Research Center for Biology, Indonesian Institute of Sciences (LIPI), Cibinong, West Java, to assess protocols for collecting and processing samples from a wide range of taxa within a single project. Here we detail our workflow for this pilot study and aim to provide practical suggestions for other researchers wishing to establish a molecular biology lab or to increase the volume of samples being handled by an existing lab. We focus on specific barriers that we identified during our pilot study at three key stages of the project: sample collection, laboratory work, and data management and processing.

Although the project we discuss here is focused on DNA barcoding of plant species, many of our recommendations apply equally to generating sequence data from other biological material. We present our results with reference to resources that describe essential infrastructure and skills needed by a small laboratory to generate high‐quality sequence data and offer suggestions for troubleshooting. Although we cannot cover every possible scenario and solution, we describe the development of a successful approach for our particular circumstances. We encourage researchers who face similar challenges to begin generating sequence data for their own research projects and applications as well as for international barcoding initiatives.

## COLLECTION OF PLANT SAMPLES

Our approach to plant collecting for this project (“A digital flora of Gunung Palung National Park”; http://www.xmalesia.info) was typical of generalist surveys of fertile plants in tropical rainforest. Unlike a subsequent project in the same locality that created permanent forest plots and then sampled and DNA‐barcoded both sterile and fertile trees (the “Xmalesia project,” also at http://www.xmalesia.info; U.S. National Science Foundation [NSF] grant no. 1020868), the project reported here sampled only fertile plants discovered opportunistically during surveys in the forest. The majority of plants included were woody trees, shrubs, and lianas, but some non‐woody herbs were also collected. Ferns were not sampled. In total, 406 specimens were collected. Of these, 371 species‐level identifications were made (with 337 unique species), 26 genus‐level identifications, and six family‐level identifications; three specimens were undetermined (Appendix S1).

### Voucher specimens

Every tissue sample taken for DNA analysis should be associated with a voucher specimen (all or part of the plant that becomes a permanent record of the specimen; see Culley, 2013). Funk et al. (2017) recently produced an excellent manual covering all aspects of voucher collection. Vouchers should be collected at least in duplicate, so that one set may be lodged in the country of origin and another set (or sets) can be sent to additional herbaria and/or the relevant taxonomic expert. Vouchers should be physical specimens except in instances where it is impossible to obtain a physical specimen; in such cases, photographic vouchers are an alternative (LaFrankie and Chua, 2015). Numerous photographs should be taken of the individual from which the voucher is made in order to record as many diagnostic features as possible (Baskauf and Kirchoff, 2008), and the vouchers themselves should also be carefully photographed. Some DNA barcoding services, such as the CCDB, offer reduced pricing if good photographic vouchers are provided. Photographic vouchers also have an additional role to play through their potential to accelerate inventories of biodiversity and support fundamental taxonomy (Webb et al., 2010).

For our project, fertile collections (of up to five duplicates) were pressed in newspaper in the field and preserved in 70% alcohol obtained from a local pharmacy. Within two weeks, they were dried in a custom‐made aluminum oven heated by a kerosene cooking stove as electricity was not available, similar to the method described in Funk et al. (2017). Dried specimens were then shipped to Herbarium Bogoriense for determination, accessioning, and distribution. For each set of duplicates, a single silica gel–dried leaf sample was also made as described below.

The collection and backup of data associated with the sampled specimens (metadata) is as important as collection of the sample itself. Detailed metadata should be recorded immediately in the field in durable field notebooks, which should themselves be photographed regularly to create a backup of the raw data. Data elements should include all the standard plant collection elements: specimen code/number, collector, date, vegetation plot code (if in plot), latitude and longitude (in decimal degrees), elevation, location, microhabitat, vegetation type, plant density (one only, a few, many), reproductive state, sex, size (height and/or diameter), plant habit (tree, liana, etc.), notable morphological features, local name, local uses, taxonomic determination in field, identity of determiner, confidence in determination (low, medium, high), and type of collection (spirit collection, carpological collection, etc.). Options now exist to enter data directly into digital devices. Although this reduces transcription errors, it can increase the chance of total loss of data. Extreme care should be taken to back up these digital records daily in the field, and/or back up to cloud storage if possible.

### Tissues for DNA extraction

Tissue for DNA extraction must be collected and processed separately from the voucher specimens described above, as described in detail by Gemeinholzer et al. (2010). Optimal tissues for DNA extraction are healthy, fully expanded leaves that are not senescing. If these leaves are large they should be torn or cut into smaller pieces to increase the drying rate (see below). If cutting, be sure to clean scissors with alcohol between specimens to prevent cross‐contamination. Although DNA extraction from cambium samples has been reported to work well (Colpaert et al., 2005), we recommend using leaf tissue because it is simple to harvest, does not require special equipment such as a cork borer, and is much less invasive. In cases where it is known that the specimens will be found as very tall trees, then preparations to take cork bores should be made, bearing in mind that obtaining permission to core trees in parks and other protected areas may be impossible. We also found that a sling shot was an excellent tool for obtaining fresh leaf material from tall trees, although great care must be taken to confirm that the fallen leaf is actually from the target tree.

The critical element when collecting tissue for DNA extraction is that it be dried rapidly because slow drying hastens DNA degradation. Rapid drying is typically achieved by placing the tissue immediately into a desiccant. A simple, effective, and economical desiccant is silica gel (Gemeinholzer et al., 2010; Neubig et al., 2014; Funk et al., 2017). The method we used for drying in silica gel is detailed in Appendix 1. Tissue can also be preserved in hexadecyltrimethylammonium bromide (CTAB) solution (reviewed in Gemeinholzer et al., 2010), or by using salt, CTAB‐salt gel, or RNA*later* (Thermo Fisher Scientific, Waltham, Massachusetts, USA; reviewed in Neubig et al., 2014) for transport back to the lab for DNA extraction.

An alternative approach is to use products such as Whatman FTA PlantSaver Cards (Whatman, Maidstone, United Kingdom; reviewed by Gemeinholzer et al., 2010; Neubig et al., 2014), where leaf squashes are made onto special paper. Care needs to be taken not to cross‐contaminate samples on the cards during collection. The paper can be used directly as a solid‐state PCR template after only a few simple washes, or the DNA can be eluted from the cards. These can be used very successfully (Siegel et al., 2017), and a similar method using Whatman paper instead of FTA cards has recently been developed and tested by Zou et al. (2017). However, as yields can be low if the DNA is eluted, and there are fewer options for troubleshooting failed PCR reactions that may stem from characteristics of the DNA sample when using the solid‐state method, paper‐based methods may be most useful for projects analyzing specimens where they have been shown to give good PCR results.

## LABORATORY PROCEDURES

### DNA extraction

Key steps leading to successful DNA extractions are grinding the tissue sufficiently and identifying the best extraction protocol(s) for the purpose at hand. The availability of lab equipment and infrastructure is also a consideration: suggestions for a minimum set of lab equipment and basic molecular biology protocols are given in Appendix 1.

Efficient grinding of the plant tissues is the first step toward high yields of DNA. For efficient and simultaneous homogenization of multiple tissue samples, we used a modified version of a grinder based on a reciprocating saw (Alexander et al., 2007; Appendix 1) as an inexpensive alternative to commercially available bead beaters. Pestles and mortars with the addition of molecular‐biology‐grade silver sand to aid grinding by hand can be used as an alternative. If a minimal sample size is needed and the tissues are soft, they can be ground in microfuge tubes with micropestles (e.g., Geneaid catalog no. MP050; Geneaid Biotech Ltd., New Taipei City, Taiwan).

The DNA extraction protocol needs to be considered carefully. Plants, especially tropical plants, synthesize a wide range of compounds, such as polysaccharides and polyphenols (Coley and Barone, 1996), that can be co‐purified with DNA and may reduce yield and/or inhibit subsequent PCR reactions. In some cases, the extraction protocol will need to be tailored to meet the specific challenges of the tissue, and it may be difficult to find a single method that works well for all samples.

Most widely used DNA extraction methods can be placed into one of two groups: those that use DNA‐binding columns to purify DNA, and those that use chemical methods to partition DNA from cellular contents in solution. DNA‐binding columns are reliable and produce consistent results, require less technical expertise to use effectively, and generate little or no hazardous waste. The major disadvantage is that they can be expensive, although cheaper versions are becoming available, and consideration is needed of the savings in time and labor achieved with kits.

If a partition‐based method is chosen, we recommend searching the literature for successes using that particular method to extract DNA from closely related taxa, or from taxa with similar extraction challenges (e.g., excess polysaccharides). There are numerous simple DNA extraction methods that have been used successfully on a variety of samples including cashew and corn (Sika et al., 2015), potato (Hosaka, 2004), Rosaceae (Antanaviciute et al., 2015), and rice (Sajib et al., 2017) that could be tested and may be successful. Otherwise, a CTAB method modified by adding agents to remove specific secondary metabolites is a good starting point; see Allen et al. (2006) and Neubig et al. (2014). Many of these methods require toxic chemicals such as phenol and chloroform, which must be handled in a fume hood and be disposed of safely in accordance with local regulations using established protocols. Safety Data Sheets (SDS) that accompany all purchased chemicals and are available online (e.g., at http://www.sigmaaldrich.com) are a good source of safety information.

**Figure 1:**
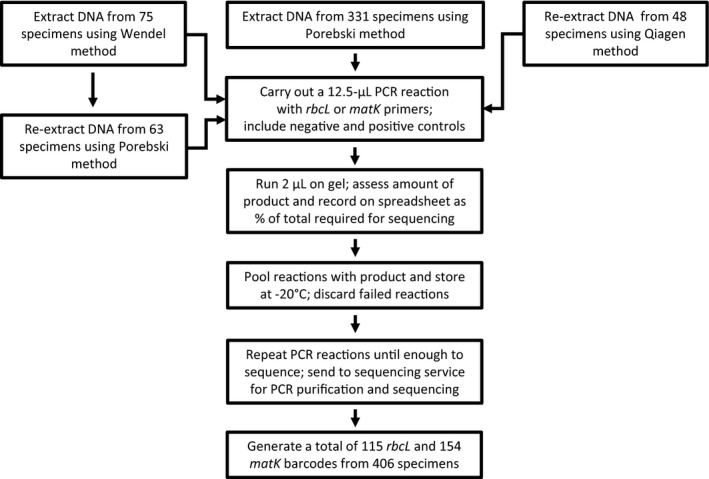
Molecular biology workflow used for processing specimens (DNA extraction and PCR amplification) during this study. DNA extraction methods used were after Tel‐Zur et al. (1999) modified by Wendel (https://www.eeob.iastate.edu/faculty/wendel/dna-extraction), Porebski et al. (1997), and the QIAGEN DNeasy Plant Mini Kit (QIAGEN, Venlo, The Netherlands).

To find the best extraction protocol for our needs, we assessed two relatively inexpensive and reasonably simple CTAB‐based methods, modified to be carried out in microfuge tubes. Both of these protocols have been used successfully by the LIPI Molecular Systematics Laboratory for taxon‐specific projects. Initially, we extracted DNA from the tissues of 75 specimens using the extraction method of Tel‐Zur et al. (1999), modified by Wendel (Appendix 1). After PCR, 63 specimens did not yield enough PCR product for sequencing both *rbcL* and *matK* (discussed in detail below). Therefore, we extracted DNA from these and a further 331 specimens, using the extraction method of Porebski et al. (1997), which generated a smaller volume of hazardous chemical waste but included one extra overnight step compared to the Wendel extraction method (Appendix 1). In total, we extracted DNA from the tissues of 406 specimens. For an additional comparison, we used the column‐based DNeasy Plant Mini Kit (QIAGEN, Venlo, The Netherlands; Appendix 1) to extract DNAs from the tissues of a subset of 48 specimens that were previously subject to CTAB extractions. The molecular biology workflow we used is shown in Figure 1, DNA extraction methods are detailed in Appendix 1, and DNA extraction data are shown in Appendix S1.

Two general approaches are widely used to determine the quantity and quality of DNA extracts. Gel electrophoresis of DNA samples and a ladder for quantification allow estimation of DNA concentrations and determination of whether the sample is degraded or contains mostly fragments of high molecular weight. Spectrophotometry allows quantity to be estimated as well as the identification of some common contaminants such as proteins and phenol. We used gel electrophoresis because we did not have access to an appropriate spectrophotometer. We attempted PCR for all samples regardless of the evidence of DNA degradation or low yield that we obtained from the gel, although lower PCR success is expected from attempts to amplify loci from DNAs that are highly degraded.

### PCR primers and amplification

Published, taxon‐specific primers for the group of interest are a good starting point for clade‐focused studies. If such primers are not available or, as is the case in our study, a wide range of taxa are being studied, universal primers designed to work across phylogenetically diverse taxa are a good option (e.g., those recommended by the CBOL Plant Working Group 2009). The criteria for CBOL‐recommended primers are based on universality (successful amplification across multiple taxa), sequence quality and coverage (amplification of regions that return high‐quality sequence data), and discrimination (enable the most species to be distinguished). Relevant taxon‐specific primer sequences can still be useful for troubleshooting if the project is broad in scope but poor PCR results are associated with particular taxa. If these approaches are not successful, primers can be designed based on publicly available sequence data. Ideally, sequence alignments should be generated from multiple taxa related to the target taxa so that suitable, conserved regions can be identified as primer sites. Primers can then be designed to amplify the region of interest using software such as PrimerDesign (Brodin et al., 2013) or Primaclade (Gadberry et al., 2005). Lorenz (2012) offers general guidelines for PCR primer design.

PCR can be challenging and, in order to achieve reproducible amplification, it is critical to use DNAs of high quality whenever possible, and to always use well‐designed primers and properly prepared and stored reagents. Storing DNA is challenging (Anchordoquy and Molina, 2007) and is discussed in detail, along with details on using frost‐free freezers for storing DNA and other reagents, in Appendix 1. Water quality is often a problem, and if reliable Milli‐Q (MilliporeSigma, Burlington, Massachusetts, USA) or equivalent water is not available, it is recommended to purchase molecular‐biology‐grade water from a reliable reagent company.

We selected PCR primers for the plant DNA barcodes *rbcL* and *matK* based on recommendations from the CBOL Plant Working Group (2009). Appendix 1 details primer sequences and PCR conditions. We performed two 12.5‐μL PCR reactions for every DNA sample extracted using a CTAB‐based protocol (Fig. 1). Two small‐volume reactions were used instead of one large‐volume reaction to give two independent attempts at amplification while conserving expensive PCR reagents. PCR products were examined using gel electrophoresis as described above. If no PCR product was generated after two attempts, no further PCRs were performed. However, if some product was present, additional PCRs were performed until there was enough DNA for sequencing. There are trade‐offs associated with performing additional PCRs to obtain enough product vs. attempting to optimize the PCR protocol for template and primer combinations that produce marginal yields. Optimization may not be practical when a project, as in this case, samples individuals from across a region or a community. When sampling closely related taxa, however, optimization could ultimately save time and resources. Suggestions for optimization and troubleshooting can be found in Appendix 1.

**Figure 2:**
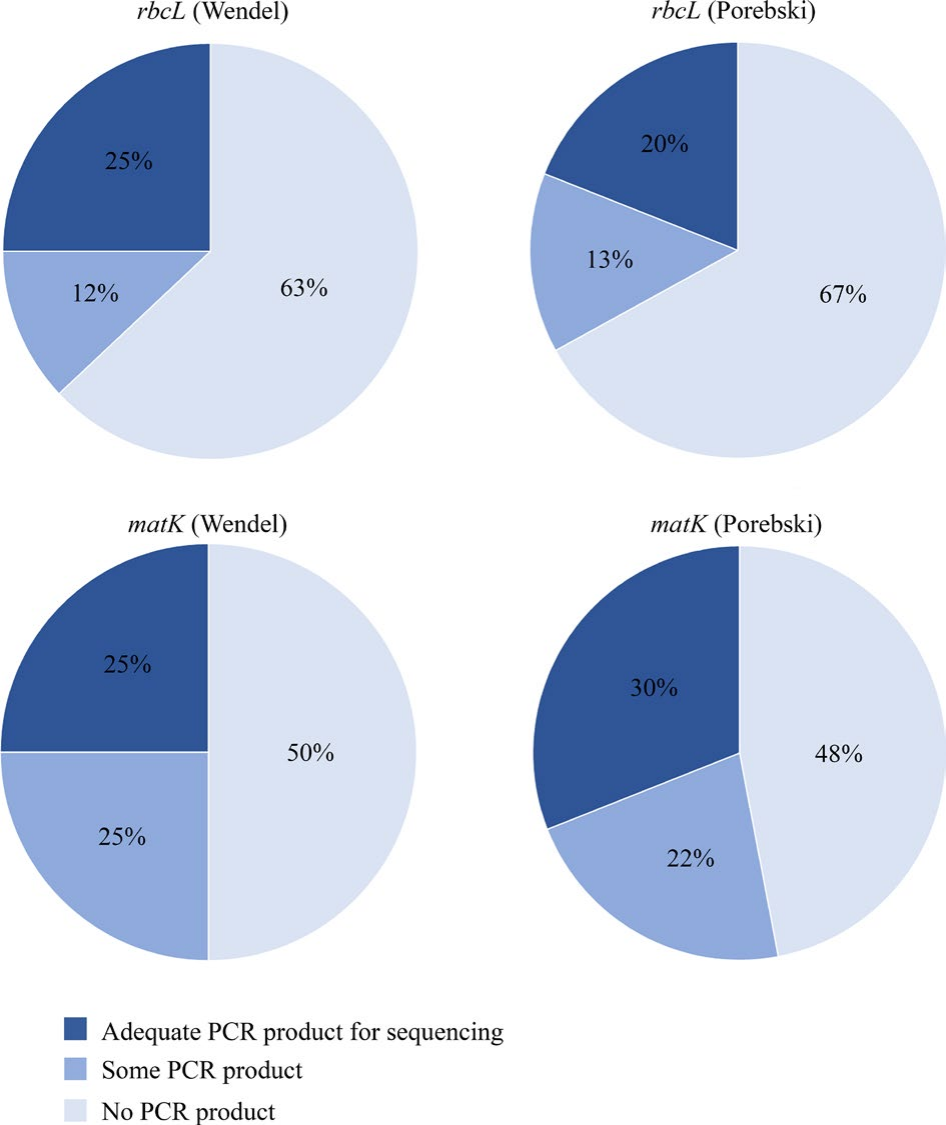
Success rates for *rbcL* and *matK* barcodes using DNA extracted using either the Porebski or Wendel CTAB methods. Yields from pooled PCR products for each extraction method were divided into three categories (no product, some product, or adequate product) and expressed as a percentage of the total number of PCR reactions performed for each combination of DNA extraction method and PCR target. DNA extraction methods used were after Tel‐Zur et al. (1999) modified by Wendel (https://www.eeob.iastate.edu/faculty/wendel/dna-extraction), Porebski et al. (1997), and the QIAGEN DNeasy Plant Mini Kit (QIAGEN, Venlo, The Netherlands).

To obtain *rbcL* barcodes, we performed up to four PCR reactions on 75 DNA samples extracted using the Wendel protocol and up to six PCR reactions on 386 DNA samples extracted using the Porebski protocol (55 samples represent extractions from specimens previously extracted with the Wendel protocol; Fig. 1). In total, we attempted to generate *rbcL* barcodes from 406 specimens (Appendix S1). We used gel electrophoresis to determine PCR yield; we assigned yields to qualitative categories in order to determine which DNA samples should be targets of additional PCR reactions to accumulate sufficient DNA for sequencing. The categories we used were “no product” when there was no visible product band; “some product” when a faint band of the expected size was visible; and “adequate product” when a bright band of the expected size was visible. These categories were based on empirical results from sequencing faint vs. bright bands. We used the same categories as described above to categorize pooled DNA from multiple PCR reactions in order to send samples for sequencing (Fig. 1). Regardless of the DNA extraction method used, we most commonly needed to carry out three or four 12.5‐μL PCR reactions to obtain enough PCR product for sequencing. Of the 75 samples extracted with the Wendel protocol, 19 were sequenced, and of the 386 samples extracted with the Porebski protocol, 76 were sequenced. A summary of these data is shown in Figure 2. A further two specimens were sequenced by pooling the PCR products from both Wendel and Porebski extractions, giving a total of 97 barcodes generated from 406 specimens (24%).

To obtain *matK* barcodes, we performed up to six PCR reactions on 73 samples extracted using the Wendel protocol and up to seven PCR reactions on 386 samples extracted using the Porebski protocol (56 samples represent extractions from specimens previously extracted with the Wendel protocol; Fig. 1). In total, we attempted to generate *matK* barcodes from 405 specimens (Appendix S1). As described above, PCR products for each extraction method were divided into three categories (no product, some product, and adequate product) based on yield estimated by gel electrophoresis. The PCR results are summarized in Figure 2. We most commonly needed to perform two (Wendel) or four (Porebski) 12.5‐μL PCR reactions per sample to obtain enough product for sequencing. Of the 73 samples extracted with the Wendel protocol, 18 were sequenced, and of the 386 samples extracted with the Porebski protocol, 116 were sequenced. A further 10 specimens were sequenced from pooled products from both Wendel and Porebski extractions, giving a total of 144 barcodes from 405 specimens (35%). Overall, the Wendel and Porebski DNA extraction methods performed similarly (Fig. 2).

**Figure 3:**
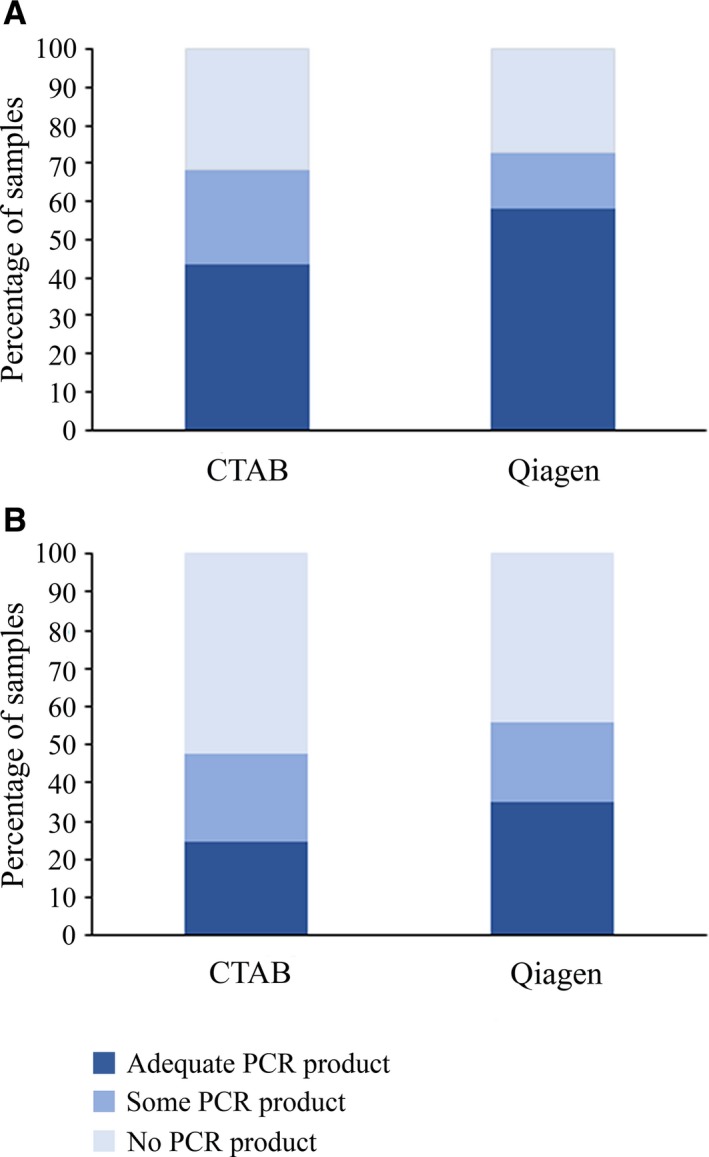
Success rates for *rbcL* (A) and *matK* (B) barcodes using DNA extracted using either CTAB‐based or QIAGEN column‐based methods. PCR products generated from a single PCR reaction using either QIAGEN‐extracted or CTAB‐extracted DNA were divided into three categories (no product, some product, or adequate product) and expressed as a percentage of the total number of PCR reactions performed for each DNA extraction method. DNA extraction methods used were after Tel‐Zur et al. (1999) modified by Wendel (https://www.eeob.iastate.edu/faculty/wendel/dna-extraction), Porebski et al. (1997), and the QIAGEN DNeasy Plant Mini Kit (QIAGEN, Venlo, The Netherlands).

The DNAs extracted using the QIAGEN DNeasy Plant Mini Kit protocol (Appendix 1) were each subject to a single PCR reaction (Fig. 1). A single PCR reaction from the corresponding CTAB‐extracted DNA was carried out at the same time. As before, PCR products were divided into three categories (no product, some product, adequate product) based on yield estimated by gel electrophoresis. The success of these single PCR reactions for *matK* and *rbcL* are shown in Figure 3, and complete details are given in Appendix S1. In terms of DNAs that could be used to generate PCR product, the QIAGEN‐extracted DNA performed similarly to the CTAB‐extracted DNA. Using the DNAs extracted using the QIAGEN kit, we generated an additional 18 *rbcL* sequences to give a total of 115/406 specimens (28%) and 10 *matK* sequences to give a total of 154/405 specimens (38%). GenBank accessions are given in Appendix S1.

Although PCR failure rates appear high, the specimens that had at least some PCR product (Fig. 2, Appendix S1) could likely be sequenced after PCR optimization to increase yield. A total of 51 specimens had some PCR product for *rbcL* (combined from all three DNA extraction methods). PCR optimization and successful sequencing of these would increase the overall success rate to 41%. Similarly, there were 75 specimens for *matK*, which if successfully sequenced, would increase the overall success rate to 57%.

As discussed above, plant taxonomic groups differ by the presence of compounds that hinder DNA extraction and amplification, and universal primers may not work for all families. Therefore, we expected our overall success to vary among plant families. We found significant association of taxonomic family with overall success of generating DNA barcodes for both *matK* and *rbcL* (respectively, *χ* ^2^ = 57.6, *df* = 11, *P* = 2.57 × 10^−8^; *χ* ^2^ = 25.5, *df* = 11, *P* = 0.00768; Table 1, Appendix S1). It is important to note the almost total failure of samples from Clusiaceae and Phyllanthaceae for both markers, and the differences in success between *matK* and *rbcL* for Annonaceae and Myristicaceae.

**Table 1:** Summary of overall success in generating DNA barcodes for *matK* and *rbcL* by taxonomic family (abundant families only)

Family^a^	*matK*		*rbcL*	
	Barcode generated	Barcode not generated	Barcode generated	Barcode not generated
Annonaceae	18	9	1	26
Apocynaceae	7	7	3	11
Clusiaceae	0	11	1	10
Dipterocarpaceae	12	8	8	12
Lauraceae	6	5	1	10
Meliaceae	7	8	6	9
Moraceae	7	14	7	14
Myristicaceae	12	2	0	14
Phyllanthaceae	1	30	7	24
Primulaceae	4	9	5	8
Rubiaceae	7	28	9	26
Other	73	120	67	127
**TOTAL**	**154**	**251**	**115**	**291**

a Families differed significantly in success rate (*matK*:* χ* ^2^ = 57.6, *df* = 11, *P* = 2.57 × 10^‐8^; *rbcL*:* χ* ^2^ = 25.5, *df* = 11, *P* = 0.00768). See Appendix S1 for full lists of success by family.

Overall, our data suggest that multiple extraction methods can be used successfully, indicating that other factors, such as kit costs, access to appropriate chemicals and infrastructure, and previous successful experience with similar samples, should be considered when choosing a method.

### Reducing contamination

Contamination can be a major problem in any molecular biology laboratory. Previously amplified PCR products are of particular concern because they may amplify much more readily than the original target locus, which may be located in a long fragment of genomic DNA. The lab should be laid out in a way that minimizes the risk of contamination. Ideally, there should be separate rooms with separate equipment and micropipettes for DNA extraction vs. PCR and all post‐PCR processes. If this is not possible, separate areas of the lab with separate micropipettes should be used for DNA extraction and PCR. Filter tips effectively reduce the amount of cross‐contamination by aerosols during pipetting and should be used if at all possible. The additional cost of filter tips is offset by reducing the generation of unusable data. Pipettes should be cleaned regularly, and fresh gloves should be worn at all times and changed frequently. It is very easy for fluids, or aerosols from fluids, to adhere to skin or gloves, and to be transferred to the next processing step. Care should be taken when handling specimens so as not to spread leaf fragments around the work area, or to cross‐contaminate samples. Forceps for sample manipulations can be sterilized by flaming or cleaned in alcohol. Negative controls (complete reaction mixes without DNA template) should be included in every set of PCR reactions to allow contamination to be detected quickly before costly sequencing is performed. The keeping of detailed records in log books on all PCR experiments is indispensable to the task of finding the source of contamination. If access to automated processing of samples is available, this presents further possibilities for reduction in contamination as well as for increasing reproducibility.

### DNA sequencing

The cost of DNA sequencing continues to decrease, and more sequencing services and platforms are becoming available. High‐throughput sequencing of barcodes (e.g., Liu et al., 2017) and metabarcoding (Deiner et al., 2017) are good options for barcoding projects that target a very high number of samples and/or ecological networks. Even whole genome shotgun sequencing at low coverage to “skim” the organellar and high‐copy nuclear loci from the sequencing reads is becoming cost‐effective (Twyford and Ness, 2017). For projects that target a small number of barcodes from specimens numbering in the hundreds to a few thousand, Sanger sequencing remains a reasonable option. The main decision is whether to outsource the sequencing of PCR‐generated barcodes, or to complete it within the institution. We recommend outsourcing to a high‐quality, affordable sequencing service as it is often cheaper than importing reagents, performing repeat reactions and troubleshooting, and maintaining instruments. Sequencing services are also in a much better position than are individual laboratories to keep up with the rapid pace of technological change in DNA sequencing approaches. Various companies offer single‐pass sequencing from as little as US$3 per sample. There are usually even greater discounts for submitting larger numbers of samples in plate format, and free shipping is available for submitting larger, but still modest, numbers of samples. Additional services such as PCR product purification are also offered by many companies, which may be more cost‐effective than importing reagents. Unlike specimens or genomic DNA, PCR products for DNA sequencing can usually be sent out of the country of origin because the samples are only a small fragment of the genome, which cannot be used for other purposes, and the sequencing reaction uses up the entire sample. We used the Sanger sequencing service at Macrogen Korea, where the requirements for sample submission were 25 μL of product at 100 ng/μL, plus 2 μL of the sequencing primer at 10 pmol/μL. Macrogen also offers a reasonably priced primer synthesis option. Shipping is free for more than 20 reactions, and one free repeat reaction is provided for failed samples, making this a very cost‐effective way to generate sequence data for a small‐scale laboratory.

High‐quality sequencing data can usually be obtained when appropriate quantity and quality standards are met, although certain sequence characteristics (e.g., high GC content and presence of simple sequence repeats) can interfere. Guidelines for quantity and quality typically are available from sequencing services, and an excellent resource for troubleshooting DNA sequence traces has been made available by the Nucleic Acid PCR Research Core Facility (NAPCore Facility, Philadelphia, Pennsylvania, USA; https://napcore.research.chop.edu/problems.php).

## DATA MANAGEMENT AND PROCESSING

### Sample data management

It is important to keep detailed log books for notes about all aspects of the laboratory work. Although the sequences generated will be submitted to publicly accessible data repositories such as GenBank (Benson et al., 2013) with electronically recorded metadata, good log books trace the history of the samples as they are processed and include all the details needed to repeat the experiments and perform troubleshooting effectively.

Spreadsheets or a database should be used to track all samples received by the laboratory, along with their metadata. Spreadsheets should be used to record which samples have been processed and their stage in the processing workflow (e.g., DNA extraction, PCR amplification, clean up, sequencing). The spreadsheet should be available to all users for addition of data as they are generated. It is essential that every user is diligent about adding their data in a timely fashion to prevent duplication, particularly when working with large numbers of samples. As with any file that is edited by several users, great care must be taken to (1) track the “master copy” and (2) make frequent backups. These issues are of less concern if shared online applications are used (e.g., Google Docs, Office 365, iCloud). However, risks associated with using a spreadsheet as a database remain, and all users should be careful to avoid these hazards (formatting and validation errors, sorting only a subset of columns and thus destroying the records’ integrity, etc.). Optimally, the spreadsheet should use data validation for all columns. Guidelines for using spreadsheets for data storage have been detailed by Broman and Woo (2017).

### DNA sequence processing and storage

After generating high‐quality sequence traces, contigs need to be trimmed, assembled, and processed. Both raw data and edited files should be stored, and everything should be regularly backed up. Although access to expensive software for processing sequence data such as DNAStar (DNASTAR Inc., Madison, Wisconsin, USA) or Sequencher (Gene Codes Corporation, Ann Arbor, Michigan, USA) can be a major obstacle for laboratories with limited funding, there exist many free (and open source) alternatives.

For viewing sequence traces in .abi format, one good free option is FinchTV (Seattle, Washington, USA), which is available for Mac, Windows, and Linux (https://digitalworldbiology.com/FinchTV). Consed, Phred, and Phrap are a free suite of programs that run on both Mac and Linux and can be used to automate base calling and quality control from sequence traces, assemble sequences, and edit sequence assemblies (Gordon et al., 1998; Ewing and Green, 1998; Ewing et al., 1998; http://www.phrap.org/phredphrapconsed.html). Additionally, there are several relatively inexpensive programs, such as ChromasPro (Technelysium, South Brisbane, Australia; Windows and Mac, used in this study) and Geneious (Biomatters Ltd., Auckland, New Zealand; Mac, Windows, and Linux), that can be used for contig assembly from traces and that allow manual editing of base calls. Geneious also includes a range of other tools for bioinformatics such as making alignments, building trees, restriction enzyme mapping, and next‐generation sequencing analysis. An excellent free program for labeling (color coding) sequence text files and restriction enzyme mapping is ApE (http://www.biologylabs.utah.edu/jorgensen/wayned/ape/; Mac and Windows).

Once the sequence data had been generated and processed, we used the Barcode of Life Database (BoLD; http://www.boldsystems.org/) to integrate and manage metadata and sequences, and we strongly recommend using this platform. BoLD facilitates sequence submission to GenBank when all of the requisite metadata for a sequence have been assembled.

## CONCLUSIONS

DNA barcoding remains a useful tool for studying biodiversity in the age of genomics (Hebert et al., 2016), for example, to provide short sequence tags for community and landscape samples (Miller et al., 2016) and to increase the efficiency of taxonomic practices (e.g., Williams et al., 2014; Wood et al., 2015). The approach is accessible to small laboratories, regardless of the scientific question at the center of the research. Currently, the main obstacles to successful generation of sequence data in resource‐limited settings are limited access to funding and training. The suggestions presented here are designed to be pragmatic and feasible in these situations and are based on our particular set of circumstances in Indonesia. The main areas for consideration are sample collection, laboratory work, and the management and analysis of sequence data.

Just as the need for locally or institutionally based sequencers has decreased or disappeared, it is likely that, in the future, more general needs for laboratory infrastructure will continue to decrease. Advances are being made in all relevant areas, including DNA amplification (e.g., isothermal PCR [Boyle et al., 2013; Tröger et al., 2015] and its incorporation into handheld devices [Tsaloglou et al., 2018]) and field‐based DNA sequencing (Parker et al., 2017), making it even easier for smaller efforts to have large in‐country impacts on biodiversity science.

## Supporting information

Appendix S1

## Appendix 1 Equipment requirements and standard techniques, sample processing, DNA extraction, and PCR primers, conditions, and troubleshooting.

### Laboratory equipment requirements and standard techniques

Standard lab equipment for DNA extraction and PCR includes, at a minimum, a microcentrifuge, water bath, pestles and mortars, micropipettes, PCR machine, gel electrophoresis apparatus, microwave, refrigerator, freezer(s), autoclave for sterilizing solutions and pipette tips, an ice machine or supply of crushed ice, and basic lab equipment to make solutions, including an analytical balance and a pH meter.

A spectrophotometer is desirable for quantifying DNA and identifying some common contaminants. A more affordable version is available from Vernier (Beaverton, Oregon, USA: http://www.vernier.com).

Freezer storage requires special consideration. Frost‐free −20°C freezers designed for household use can be unsuitable for storing molecular biology reagents because they usually have time‐based auto‐defrost cycles that may allow the contents of the freezer to thaw in addition to removing frost build‐up from inside the freezer. Specialized laboratory frost‐free freezers have temperature‐sensitive auto‐defrost cycles to remove frost build‐up that also prevent the contents from defrosting, but these are much more expensive. Possible solutions are to choose freezers that need to be manually defrosted, or to invest in freezer boxes that contain a coolant that remains frozen during the defrost cycle (e.g., Thermo Scientific Nunc catalog no. 355501; Thermo Fisher Scientific, Waltham, Massachusetts, USA).

Storage of DNA is a challenging problem (Anchordoquy and Molina, 2007). Template DNA is best stored in buffer at slightly alkaline pH (e.g., Tris buffer at pH 8.0), and although including EDTA is beneficial for DNA stability (e.g., TE‐8 buffer), it may inhibit the activity of DNA polymerase (Neubig et al., 2014). If template DNA is stored in buffer with EDTA, then the samples should be diluted in water before use. Working DNA solutions that are used regularly (e.g., template DNA, primers, and dNTPs) can be stored at −20°C (template DNA) or −80°C (best for primers and dNTPs) in small aliquots to avoid repeated cycles of freezing and thawing that cause degradation (Davis et al., 2000; Schaudien et al., 2007). For DNA templates, it is wise to have a working solution that is used regularly for setting up PCR, and to keep a long‐term stock that is not thawed or opened on a regular basis. For long‐term storage, the colder the better (liquid nitrogen is best, followed by −80°C, then −20°C; Neubig et al., 2014). Storage of DNA on FTA cards as described above allows stable room temperature storage, and there are other possibilities to store extracted DNA at room temperature (e.g., if it is vitrified using trehalose [reviewed in Neubig et al., 2014]).

An excellent resource for standard molecular biology laboratory techniques is Cold Spring Harbour Protocols (http://www.cshprotocols.cshlp.org), and BioProtocol (http://www.bio-protocol.org) has methods for more specialized techniques. The Questions section of ResearchGate (http://www.researchgate.net) is also a very useful resource.

#### Sample desiccation—

A simple, effective, and economical desiccant is silica gel (Gemeinholzer et al., 2010; Neubig et al., 2014; Funk et al., 2017). Fine floral silica works extremely well, but it presents an inhalation hazard and was hard to find in Indonesia at reasonable prices. Instead, we used indicating silica gel (handled with care as the indicator is toxic cobalt chloride) with a bead size of approximately 3 mm. Nontoxic indicators such as those based on iron III/II salts are also available. We found that placing silica into bags alongside samples made it difficult to change the beads for fresh silica without losing plant material, because leaves break up as they dry. Samples in paper envelopes did not dry rapidly enough, so we switched to tea bags (Wilkie et al., 2013). We used unbleached 120 × 87‐mm bags from Danske Tefilter AS (Copenhagen, Denmark), but any similar local product would be suitable. Samples in tea bags were placed in airtight boxes with a large excess of silica, making sure that the bags were separated with silica to enable quick drying. The silica was checked every day and exchanged as required until the samples were dry. Once dry, samples were stored in airtight boxes to maintain desiccation, which requires less silica gel than during the drying phase. Make sure that the label on the silica samples captures enough information to link it to the voucher specimen (collection number, date, collector's name). Once the samples are dried and packaged in this way, it is necessary to be vigilant during storage in the laboratory. Airtight boxes containing samples and silica were checked monthly and silica exchanged promptly when needed. Funk et al. (2017) and Gemeinholzer et al. (2010) contain detailed information on sample collection for DNA extraction.

#### Tissue disruption—

Silica‐dried tissue (20 mg) was added to labeled 2‐mL microfuge tubes containing MP Biomedicals Lysing Matrix A (catalog no. 116910; MP Biomedicals, Santa Ana, California, USA). Beads were reused with a pinch of sand in place of Lysing Matrix A (e.g., catalog no. 274739; Sigma‐Aldrich, St. Louis, Missouri, USA). Tissue was ground using a reciprocating saw–based grinder (Alexander et al., 2007). Tubes were placed symmetrically into a rack for microfuge tubes attached to the reciprocating saw and secured with electrical tape. After affixing the attachment properly and securely to the reciprocating saw (with safety glasses and lab coat on), the saw was held with both hands in a vertical position pointing toward the floor. To check that the rack was balanced correctly, the power button was partially depressed. If any wobbling occurred, the rack was rebalanced. If the rack was balanced, the power button was depressed the entire way for 30–60 s (grinding for more than 1 min can crack the lid and/or tube). After grinding, DNAs were extracted using one of the three following DNA extraction methods.

#### DNA extraction after Tel‐Zur et al.—

This method was first described by Tel‐Zur et al. (1999) and then modified by Wendel (https://www.eeob.iastate.edu/faculty/wendel/dna-extraction). The protocol below contains additional notes and modifications from our lab.

##### Reagents and solutions—

- Extraction buffer: 100 mM Tris‐HCl (pH 8.0), 0.35 M sorbitol, 5 mM ethylenediaminetetraacetic acid (EDTA, pH 8.0), and 1% 2‐mercaptoethanol (added just before use). Chill on ice.
- High‐salt CTAB buffer: 50 mM Tris‐HCl (pH 8.0), 4 M NaCl, 1.8% w/v cetyltrimethylammonium bromide (CTAB), and 25 mM EDTA (pH 8.0)
- Sarkosyl (30% w/v in water)
- Chloroform : isoamyl alcohol (24 : 1)
- Isopropanol (100%)
- TE buffer (10 mM Tris‐HCl, 1 mM EDTA, pH 8.0)
- RNase A (10 mg/mL in water)
- Sodium acetate (3 M, pH 5.2)
- Phenol
- Phenol : chloroform (1 : 1; make just before use)
- Chloroform
- Ethanol (70% and 100%, both ice cold)

##### Protocol—

1. Add 2 mL of ice cold extraction buffer to 20 mg of dried, ground tissue in a 2‐mL microfuge tube prepared as described above.
2. Gently mix tubes by inversion for 5 min.
3. Centrifuge tubes at 5000 × *g* at 4°C for 10 min.
4. Remove the supernatant to a new 2‐mL microfuge tube, add another 2 mL of ice cold extraction buffer and wash by inversion for 5 min.
5. Centrifuge tubes at 5000 × *g* at 4°C for 10 min.
6. Remove the supernatant to a new 2‐mL microfuge tube, add 0.5 mL of ice cold extraction buffer, and mix briefly and gently.
7. Add 350 μL of high‐salt CTAB buffer and 30 μL of 30% sarkosyl. Incubate on a shaker at room temperature at 50–60 rpm for 1 to 1.5 h.
8. Add an equal volume of chloroform : isoamyl alcohol (approximately 880 μL).
9. Mix gently and then centrifuge at 5000 × *g* for 10 min. Three layers will form: an upper phase (aqueous phase), a thin interphase, and a lower phase. Transfer the upper phase that contains DNA into a new 1.5‐mL microfuge tube, being very careful not to remove any of the interphase or lower phase.
10. Add 2/3 (v/v) cold isopropanol (approximately 600 μL) and invert several times. This is a possible stopping point where the samples can be stored at 4°C overnight.
11. Centrifuge at 5000 × *g* for 10 min, then decant the solution, leaving the pellet behind.
12. Add 1 mL of cold 70% ethanol and swirl to mix.
13. Centrifuge at 5000 × *g* for 5 min, then decant the solution, leaving the pellet behind.
14. Dry the pellet for 30 min upside down on paper towel on the bench.
15. Add 300 μL of TE buffer and dissolve pellet in water bath (up to 60°C) for 15 min.
16. Add 3 μL of RNase A and incubate at 37°C (or room temperature) for 60 min.
17. Add 300 μL of phenol, thoroughly mix by inversion or gentle vortexing, and centrifuge at 10,000 × *g* for 10 min.
18. Transfer the upper (aqueous) phase to a new 1.5‐mL microfuge tube. Use the same caution as in step 9.
19. Add 300 μL of 1 : 1 phenol : chloroform, thoroughly mix by inversion or gentle vortexing, and centrifuge at 10,000 × *g* for 10 min.
20. Transfer the upper (aqueous) phase to a new 1.5‐mL microfuge tube. Use the same caution as in step 9.
21. Add 300 μL of chloroform, thoroughly mix by inversion or gentle vortexing, and centrifuge at 10,000 × *g* for 10 min.
22. Transfer the upper (aqueous) phase to a new 1.5‐mL microfuge tube. Be extremely careful not to pipette up any of the interphase or lower phase at this specific extraction!
23. Add 2 volumes of ice cold 100% ethanol (approximately 600 μL) combined with 1/10 volume sodium acetate (approximately 30 μL). Store at −20°C for 30 min, or overnight if convenient.
24. Centrifuge the tubes at the highest speed for 15 min to pellet the DNA. Decant the supernatant.
25. Add 1 mL of ice cold 70% ethanol and leave at room temperature for 5 min.
26. Centrifuge at 5000 × *g* for 5 min and decant the supernatant.
27. Dry the pellet for 30 min upside down on paper towel at the bench.
28. Dissolve the pellet in 15 μL of TE.

#### DNA extraction after Porebski et al.—

This protocol was adapted from Porebski et al. (1997) to be carried out in microfuge tubes.

##### Reagents and solutions—

- Extraction buffer: 100 mM Tris‐HCl (pH 8.0), 1.4 M NaCl, 20 mM EDTA (pH 8.0), 2% (w/v) CTAB, and 0.3% 2‐mercaptoethanol (added just before use)
- Polyvinylpyrrolidone (PVP)
- Chloroform : isoamyl alcohol (24 : 1)
- Sodium chloride (5 M)
- Ethanol (100% and 70%, both ice cold)
- TE‐8.4 buffer (10 mM Tris‐HCl, 1 mM EDTA, pH 8.4)
- RNase A (10 mg/mL in water)
- TE‐8 buffer (10 mM Tris‐HCl, 1 mM EDTA, pH 8)
- Sodium acetate (2 M, pH 5.2)

##### Protocol—

1. Add 500 μL of extraction buffer (preheated to 60°C) and 5 mg of PVP to 20 mg of dried, ground tissue in a 2‐mL microfuge tube prepared as described above.
2. Mix by inversion and incubate at 60°C with shaking (or mix regularly) for 60 min.
3. Cool to room temperature for 5 min, then add 600 μL of 24 : 1 chloroform : isoamyl alcohol.
4. Mix by inversion and centrifuge at 1000 × *g* for 20 min. Three layers will form: an upper phase (aqueous phase), a thin interphase, and a lower phase. Transfer the upper phase that contains DNA into a new 1.5‐mL microfuge tube, being very careful not to remove any of the interphase or lower phase.
5. Add 1/2 volume (approximately 225 μL) of sodium chloride and mix well.
6. Add 2 volumes (approximately 900 μL) of 100% ethanol and mix well.
7. Leave to precipitate at 4°C overnight.
8. Centrifuge at 1000 × *g* for 6 min, then decant the solution, leaving the pellet behind.
9. Add 1 mL of ice cold 70% ethanol and leave at room temperature for 5 min.
10. Centrifuge at 1000 × *g* for 6 min, then decant the solution, leaving the pellet behind.
11. Dry the pellet for 30 min upside down on paper towel at the bench.
12. Dissolve the pellet in 200 μL of TE‐8.4 overnight at 4°C.
13. Add 2 μL of RNase A and incubate at 37°C (or room temperature) for 60 min.
14. Add 200 μL of phenol, thoroughly mix by inversion or gentle vortexing, and centrifuge at 20,000 × *g* for 15 min.
15. Transfer 150 μL of the upper (aqueous) phase to a new 1.5‐mL microfuge tube. Use the same caution as in step 4.
16. Add 50 μL of TE‐8.4 to the phenol phase, mix by inversion or gentle vortexing, and centrifuge at 20,000 × *g* for 15 min.
17. Transfer 50 μL of the upper phase (using same caution as in step 4) to the 1.5‐mL microfuge tube containing the upper phase from the first extraction.
18. Add 2 volumes of ice cold 100% ethanol (400 μL) combined with 1/10 volume sodium acetate (20 μL). Store at –20°C for 30 min, or overnight if convenient.
19. Centrifuge at 20,000 × *g* for 20 min and discard the supernatant.
20. Add 1 mL of ice cold 70% ethanol and leave at room temperature for 5 min.
21. Centrifuge at 20,000 × *g* for 5 min and discard the supernatant.
22. Dry the pellet for 30 min upside down on paper towel at the bench.
23. Dissolve the pellet in 30 μL of TE‐8.

#### DNeasy Plant Mini Kit (QIAGEN) DNA extraction—

##### Reagents and solutions—

All solutions are provided as part of the kit, apart from 100% ethanol that is added to buffer AP3 before use.

##### Protocol—

1. Add 400 μL of Buffer AP1 and 4 μL of RNase A stock solution to 20 mg of dried, ground tissue in a 2‐mL microfuge tube prepared as described above.
2. Vortex vigorously, then incubate for 60 min at 65°C with shaking (or mix regularly).
3. Centrifuge for 1 min at 2500 × *g*, then transfer the supernatant to a new 1.5‐mL microfuge tube.
4. Add 130 μL of Buffer AP2 and incubate on ice for 15 min.
5. Centrifuge at 20,000 × *g* for 5 min, then transfer the supernatant to a QIAshredder spin column (lilac) sitting in a 2‐mL collection tube.
6. Centrifuge at 20,000 × *g* for 2 min.
7. Transfer the flowthrough (containing the DNA) to a new 1.5‐mL microfuge tube, being careful not to disturb any pellet. 450 μL of lysate is usually recovered, but it could be less for some samples; measure the volume exactly using a micropipette.
8. Add 1.5 volumes of Buffer AP3 (pre‐mixed with ethanol according to kit instructions), e.g., 675 μL of Buffer AP3 for 450 μL of lysate, and mix gently by pipetting up and down.
9. Transfer 650 μL of this mixture, including any precipitate that may have formed, to a DNeasy Mini Spin Column (white) sitting in a 2‐mL collection tube.
10. Centrifuge at 6000 × *g* for 1 min.
11. Discard the flowthrough, then add the remainder of the sample to the same DNeasy column and centrifuge at 6000 × *g* for 1 min.
12. Discard the collection tube and flowthrough and place the column into a new collection tube.
13. Wash the DNA bound to the column by adding 500 μL of Buffer AW and centrifuge at 6000 × *g* for 1 min.
14. Discard the flowthrough and wash the column again with a further 500 μL of Buffer AW as described above.
15. Discard the flowthrough, then dry the column by centrifuging for 2 min at 20,000 × *g*.
16. Transfer the DNeasy column to a new, labeled 1.5‐mL collection tube, being careful not to contact the flowthrough and carry over any ethanol.
17. Elute the DNA with 75 μL of Buffer AE (preheated to 65°C) pipetted directly onto the DNeasy column membrane.
18. Let the column stand for 1–5 min at room temperature, then centrifuge at 6000 × *g* for 1 min.
19. Repeat the elution with another 50 μL of preheated Buffer AE as described above. Collect the second elution in the same tube as the first elution.

#### PCR primers and conditions—

PCR mixtures contained the following reagents: 1× Green GoTaq Flexi Buffer, 0.2 mM dNTP mix (Promega catalog no. U1511; Promega Corporation, Madison, Wisconsin, USA), 0.35 units of GoTaq Flexi DNA Polymerase (Promega catalog no. M8291), 0.2 mM (*rbcL*) or 0.48 mM (*matK*) of each primer, 2.5 mM of MgCl_2_, 1 μL of DNA, and water to a total volume of 12.5 μL. DNA was diluted in water before use (one in 10 for CTAB extraction protocols, and one in two for the QIAGEN extraction method).

For *rbcL*, the primers were rbcL_1f 5′‐ATGTCACCACAAACAGAAAC‐3′ and rbcL_724r 5′‐TCGCATGTACCTGCAGTAGC‐3′ from Fay et al. (1997) with cycling conditions of 94°C for 4 min; five cycles of 94°C for 30 s, 55°C for 1 min, 72°C for 1 min; 30 cycles of 94°C for 30 s, 54°C for 1 min, 72°C for 1 min; 72°C for 10 min; 12°C hold.

For *matK*, the primers were 3F_KIMf 5′‐CGTACAGTACTTTTGTGTTTACGAG‐3′ and 1R_KIMr 5′‐ACCCAGTCCATCTGGAAATCTTGGTTC‐3′ from Ki‐Joong Kim (Department of Life Sciences, Korea University, Seoul, South Korea; unpublished data) with cycling conditions of 94°C for 5 min; 35 cycles of 94°C for 30 s, 55°C for 45 s, 72°C for 1.5 min; 72°C for 10 min; 12°C hold.

#### PCR troubleshooting—

For failed samples, we make the following suggestions. Positive controls should be included to ensure that the PCR components are functional. Altering the amount of DNA in the PCR reaction can be helpful; diluting DNA also dilutes impurities that may inhibit PCR, and conversely, increasing DNA concentration may increase the amount of template to the required threshold level. It is simplest to set up a concentration curve, using different amounts of DNA (prepared as a serial dilution) to determine the optimal amount. Adding DNA suspected of containing inhibitors to a reaction that is reliably successful can be used to test for presence of inhibitors. Magnesium concentration can also affect PCR success. Determine the optimum concentration of magnesium by performing a set of PCR reactions with different concentrations; choose the lowest concentration that works, as higher magnesium concentration may lead to loss of fidelity of some DNA polymerases. Similarly, optimize the annealing temperature by performing PCR reactions with different annealing temperatures (simultaneously on a gradient PCR block if possible); choose the one with the greatest amount of target PCR product and the least evidence of nonspecific bands. Re‐extracting the DNA using a different method is also an option, especially if the method is designed to remove species‐specific impurities that might inhibit enzyme activity. Finally, the primers can be changed or redesigned for the target in specific taxa. Lorenz (2012) is a good source of detailed information on setting up PCR reactions, as well as suggestions for troubleshooting.
